# Nuclear translocation of Atox1 potentiates activin A-induced cell migration and colony formation in colon cancer

**DOI:** 10.1371/journal.pone.0227916

**Published:** 2020-01-21

**Authors:** Arundhati Jana, Archita Das, Nancy L. Krett, Grace Guzman, Alexandra Thomas, Georgina Mancinelli, Jessica Bauer, Masuko Ushio-Fukai, Tohru Fukai, Barbara Jung

**Affiliations:** 1 Division of Gastroenterology and Hepatology, University of Illinois Medical College, Chicago, IL, United States of America; 2 Vascular Biology Center, Department of Pharmacology and Toxicology, Medical College of Georgia at Augusta University, Augusta, GA, United States of America; 3 Department of Pathology, University of Illinois Medical College, Chicago, IL, United States of America; 4 Vascular Biology Center, Department of Medicine (Cardiology), Medical College of Georgia at Augusta University, Augusta, GA, United States of America; 5 Charlie Norwood Veterans Affairs Medical Center, Augusta, GA, United States of America; Duke University School of Medicine, UNITED STATES, United States of America

## Abstract

**Background:**

Colorectal cancer remains a deadly cancer due to metastatic disease. To understand the molecular mechanisms of metastasis in colon cancer, we investigated whether the copper chaperone antioxidant-1 (Atox1) protein plays a role in this process. Recent findings indicate that Atox1 protein has transcription factor activities and plays a vital role in cell proliferation in cancer cells. However, the role of Atox1 in metastasis has not been examined.

**Methods:**

Atox1 expression was determined by immunofluorescence in a tissue microarray generated from a spectrum of CRC patients. Subcellular fractionation of colon cancer cell lines SW480 and SW620 cells was used to examine the cellular location of Atox1 in the face of activin A, a cytokine that stimulates colon cancer metastasis. Atox1 expression was genetically manipulated and cellular migration measured through trans-well assay and proliferation measured by colony formation assays.

**Results:**

Here we demonstrate that in patients with metastatic colon cancer, there is a significant increase in the expression of nuclear Atox1. Interestingly, the metastatic CRC cell line SW620 has increased nuclear localization of Atox1 compared to its related non-metastatic cell line SW480. Further, inhibition of endogenous Atox1 by siRNA in SW620 decreased colony formation and reactive oxygen species generation via decreased expression of Atox1 targets cyclin D1 and NADPH oxidase subunit p47 phox, respectively. Additionally, overexpression of nuclear-targeted but not copper binding domain-mutated Atox1 in SW480 cells increased colony formation and cell migration that was further augmented by activin A stimulation, a known enhancer of colon cancer metastasis.

**Conclusions:**

Our findings suggest that nuclear Atox1 might be a new therapeutic target as well as a new biomarker for metastatic colorectal cancer.

## Introduction

Colorectal cancer (CRC) is a common and deadly cancer due to its metastatic nature [1]. Although many breakthroughs in the diagnosis and treatment of CRC have been made over the past decades, distant metastasis remains the major cause of CRC-related mortality [2–4]. Though the localized forms of CRC can be effectively managed, no curative treatment is currently available for metastatic CRC [2–4]. While tumor metastasis is a complicated process, the TGFβ family member activin A is known to play a crucial role in promoting CRC metastatic actions [5]. Overexpression of activin A is more pronounced in stage IV colorectal cancer, and activin A stimulates tumor cell migration and epithelial to mesenchymal transition (EMT)[6–8]. Although several biomarkers have been associated with prognosis in CRC, prognostic prediction of metastatic CRC is still lacking. Hence, it is of great clinical value to identify novel biomarkers which could be utilized as possible therapeutic targets to optimize the treatment of patients with metastatic CRC.

Antioxidant protein 1 (Atox1) is known to play a key role in copper homeostasis [9]. It regulates the intracellular concentrations of copper by transporting the cytosolic copper (Cu) that has entered the cell through the membrane-bound Cu importer CTR1 to Cu exporter ATP7A and/or ATP7B located in the trans-Golgi network secretory pathway, thereby preventing copper toxicity [10]. Intriguingly, in addition to its canonical Cu chaperone function, Atox1 has been reported to play an important role in angiogenesis and wound repair [11, 12]. Thus, the function and regulation of Atox1 and its role in cancer have attracted much attention. Atox1 was highly expressed in a spectrum of cancers [13–16]. Interestingly, Atox1 protein has been reported to behave as a Cu-dependent transcription factor whose function requires a Cu binding domain (CBD) and a C-terminal conserved lysine-rich region that acts as the nuclear localization signal (NLS) [17–19]. Atox1 has been reported to promote the expression of cyclin D1 and NADPH oxidase p47 phox expression leading to increased proliferation and ROS generation [11, 12, 18]. However, the expression pattern and role of Atox1 in metastatic CRC remains largely unknown. Therefore, we studied the link between Atox1 and activin A in CRC metastasis.

The present study explored the role of Atox1 in CRC using metastatic and non- metastatic CRC cell lines and a patient tissue microarray (TMA). Interestingly, we found that Atox1 nuclear location was significantly correlated with the severity of metastatic CRC in a TMA of colorectal tumors from patients. Consistent with this, Atox1 is highly expressed in the nucleus in metastatic SW620 colon cancer cell line, while Atox1 is expressed more in the cytosol in non-metastatic SW480 colon cancer cell lines. Activin A treatment promotes Atox1 nuclear translocation in both colon cancer cell lines. Endogenous inhibition of Atox1 in metastatic SW620 human colon cancer cells decreased cellular migration, viability and colony formation, which was associated with decrease in cyclin D and p47 phox expression. Importantly, we found that nuclear-targeted Atox1 induces migration and colony formation in non-metastatic SW480 colon cancer cells that was further increased upon activin A stimulation. Our findings represent a functional role for Atox1 in CRC metastasis.

## Materials and methods

### Reagents

The nuclear-targeted Atox1 plasmid (Flag-Atox1-NLS) construct containing a tripartite NLS (nuclear localization signal) sequence (PKKKRKVD) was derived from the SV40 large T antigen fused to the C terminus of Flag-tagged w-Atox1 (wild type) as previously described [18]. The Atox1 CBD (copper binding domain) has a mutated copper binding domain (Atox1 C12,15 S).

### Tissue microarray (TMA)

The human colorectal carcinoma tissue array study was approved by the University of Illinois at Chicago (UIC) Institutional Review Board and tissues collected only after written informed consent was administered and signed. There are no individual case notes or any patient private health information included in this manuscript. Briefly, tumor tissue derived from colon was obtained from patients diagnosed with colorectal carcinoma at the University of Illinois Medical Center. A total of sixty colorectal resection specimens cores from the formalin fixed paraffin embedded tumor were placed in a tissue micro-array. The quality and structural integrity of the tissue blocks were appraised and evaluated before inclusion into the study as described previously [20]. A total of four tissue array recipient blocks were created comprising of duplicate 0.6 mm diameter tissue cores representing normal colonic mucosa, hyperplastic colonic mucosa, dysplastic colonic mucosa, and colonic adenocarcinoma. Four-micron thick tissue array sections were placed on positively charged glass slides for immunofluorescence studies [21].

### Immunofluorescence

Formalin-fixed and paraffin-embedded TMAs were de-paraffinized in xylene and then rehydrated in descending ethanol series. The sections were blocked in 3% BSA for 60 min to block nonspecific binding sites. Immunostaining was performed at 4°C overnight with a rabbit Atox1 antibody (1:500 dilution)[18]. Following primary antibody, the TMAs were washed three times with PBS and incubated with appropriate secondary antibody for 1h at room temperature and then stained with 4,6-diamidino-2-phenylindole (DAPI). Finally, the sections were passed through graded alcohol and mounted. Slides were observed under fluorescent microscope. For negative controls, a TMA slide was incubated under similar conditions without the primary antibodies.

### Scoring of immunofluorescence staining result

The colon cancer specimens on the TMA slides were examined and scored. A staining index for each tissue core was obtained based on published methods [21, 22]. The immuno-fluorescence staining intensity of the nuclei was assessed using a three-tier grading system. Staining intensity was assessed (weak or no staining = 0, moderate staining = 1, strong staining = 2) as well as the extent of stained nuclear positive cells (0–10% = 0.5, 10–50% = 1, 50–100% = 2). The physical characteristics of the tumor, background pathology, and patient demographic information were recorded. The final immunoreactive score of nuclear Atox1 was determined by multiplying the intensity scores with the extent of positivity scores for nuclei. Slides were scored in a blinded fashion by two investigators. Both investigators had to be in agreement for a tumor to be called negative.

### Colon cancer cell lines

SW480 and SW620 cell lines were acquired from the American Type Culture Collection (ATCC). The cell lines were cultured in a 5% CO_2_ chamber supplemented with 10% FBS and 1% Pen Strep containing media and were serum starved for 24 hours prior to treatment. To limit variability between experiments, cells were cultured to a maximum of 30 passages to minimize mutations in the cell lines. In addition, cell cultures under identical conditions of treatment were done in parallel to validate our findings. Further, all the cell lines were tested for mycoplasma prior to starting experiments. The cells were authenticated once a year and validated by 9 STR (short tandem repeat) profiling using Cell Check 9Plus and tested for mycoplasma (both IDEXX, Columbia, MO, USA).

### Western blot analysis

Cell lysates were treated with bicine chaps buffer containing 1x protease cocktail inhibitor (Roche, USA) and prepared for western blot analysis as previously described [21]. Blots were immunoblotted with different antibodies including Atox1 (1:1000), GAPDH (1:500 dilution), p84 (1:500 dilution), Cyclin D1 (1:500 dilution) and p47 phox (1:500 dilution) (Cell Signaling Technology, MA, USA) overnight at 4°C. After washing three times in TBST buffer, the membranes were exposed to HRP-conjugated secondary antibodies for 1 hour at room temperature and imaged using enhanced chemiluminescence to detect immobilized antibodies. Densitometric analysis of immunoblots for respective proteins (Atox1, GAPDH, Nuclear matrix protein (p84), Cyclin D and p47 phox) was done by using image J software. All the experiments were repeated at least three times under same conditions.

### Subcellular fractionation

Colon cancer cell lines were subjected to subcellular fractionation using NE-PER nuclear and cytoplasmic extraction reagents (Thermofisher Scientific, CO, USA) according to manufacturer’s instruction. Fractionation efficiency was determined by western blot analysis using GAPDH as cytoplasmic and p84 as nuclear protein controls respectively.

### Transfection

Colon cancer cells were transfected with 1 ug of plasmid DNA encoding Flag tagged WT Atox1, Atox1 NLS, Atox1 CBD using lipofectamine 3000 in 12-well plates at a density of 1x10^6^ cells according to the manufacturer’s instruction. After 48 hours of transfection cells were left either untreated or treated with activin A (Ansh Labs, TX, USA) for an additional 24 hours. Protein expression of transfected cDNAs was confirmed by western blot analysis. Specific siRNA for Atox1 (Ambion, TX, USA) was transiently delivered at a final concentration of 30 nM via electroporation using AMAXA Nucleofector (Lonza, Basel, Switzerland). Transfection efficiency was confirmed by fluorescent microscope using the pmaxGFP control vector. Forty-eight hours post transfection cell lysates were prepared for western blot analysis.

### Migration assay

Colon cancer cell lines (5x10^4^ cells/well) were plated into Transwell 12 well plates (8μm pores, Corning, NY, USA) that were coated with fibronectin (Sigma) to facilitate migration. After 6 hours, non-migrated cells at the membrane top were removed and the cells that have migrated through the membrane were fixed with 4% PFA and stained with DAPI (nuclear stain). Images were captured by fluorescent microscopy and the number of migrated cells per well was determined by counting the cells at the center of each well using image J software to digitized images. Data are expressed as number of particles counted per high powered field (hpf).

### Cell viability assay

Mitochondrial activity as a surrogate for cell number and was measured with cell counting kit-8, (CCK8, Dojindo Molecular Technologies, MD, USA) as previously described [5]. The cells were grown at a density of 5x10^3^ cells/well on 96-well culture plates with 100 ul of medium and treated with various reagents as outlined in the figure legends. The assay was developed as per the manufacturers protocol.

### Colony formation assay

For colony formation assay, cells were seeded into six-well plates at a density of 100 cells per well and incubated for two weeks in DMEM containing 10% FBS at 37°C. After two weeks, colonies were fixed by cold methanol for 15 minutes and stained with 0.5% crystal violet for 1 hour. The number of colonies (>50cells/colony) was counted using a stereomicroscope and analyzed by image J software [23]. All the samples were done in triplicate.

### Statistics

All data are expressed as means ± SD from at least three independent experiments. Statistical analyses for differences were performed via Student’s t test when two groups are analyzed and by one-way ANOVA followed by Newman-Keul’s multiple comparison analysis using Graphpad Prism 5.0 when more than two groups were analyzed. The criterion for statistical significance was p > 0.05.

## Results

### Nuclear enrichment of Atox1 in patients correlated with metastatic CRC

**Fig 1:**
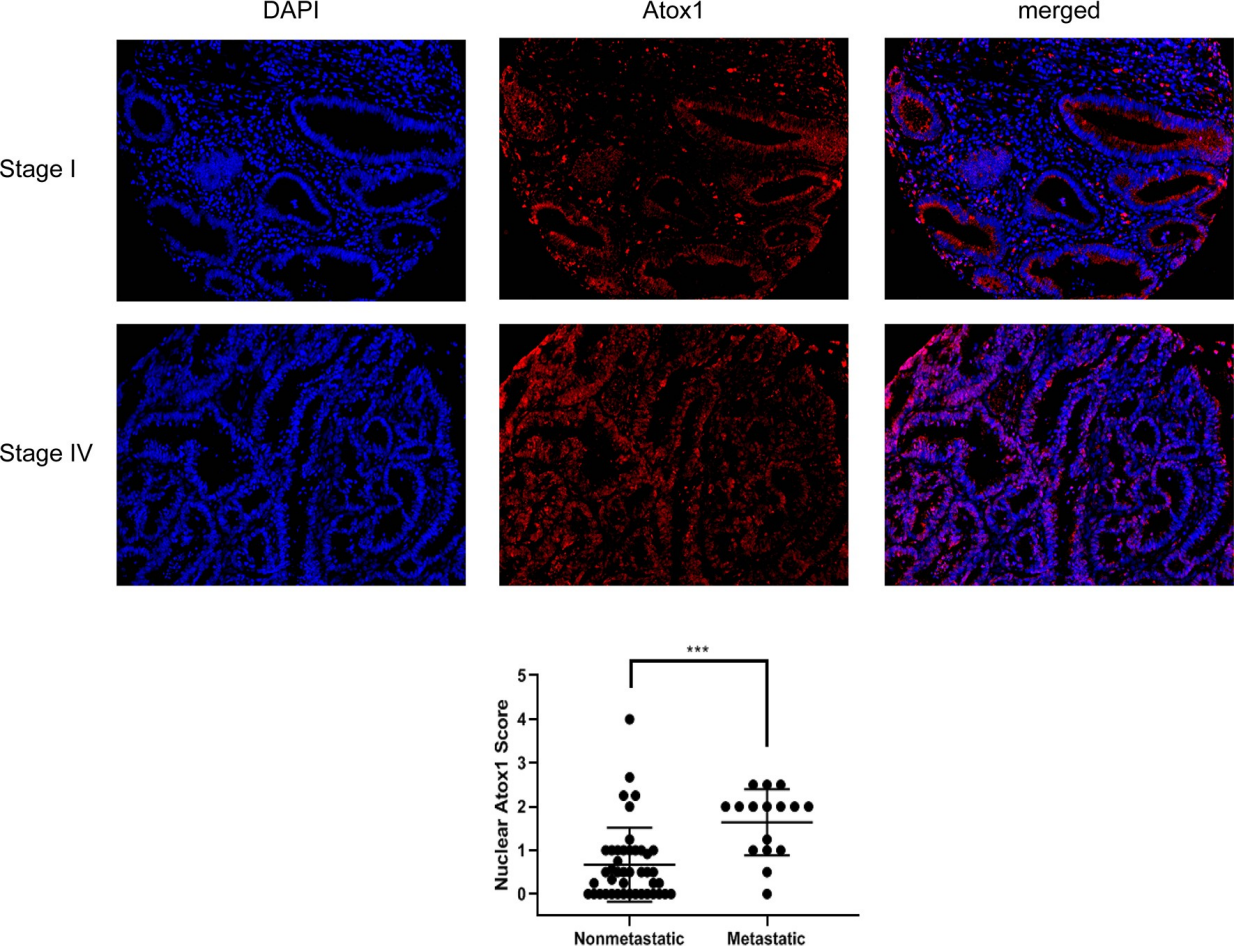
Nuclear Atox1 expression is greater in metastatic colon cancer. A TMA consisting of colonic adenocarcinomas and adjacent normal colonic mucosa from 60 colorectal cancer patients was stained for Atox1. Immunofluorescence staining was used to examine the expression level and localization of Atox1. The red color indicates Atox1 and blue color indicates DAP1. The nuclear staining score for Atox1 for each patient section was calculated as described in Materials and Methods and is graphed (p<0.001) comparing staining from tissue sections of non-metastatic disease to metastatic disease.

To characterize the role of endogenous Atox1 in CRC metastasis, immunofluorescent staining of Atox1 protein was examined in a tissue microarray (TMA) consisting of adenocarcinoma and adjacent normal epithelial mucosa from 60 CRC patients (Fig 1). We observed immunoreactivity of Atox1 protein in both nuclear and cytoplasmic compartments of colon cancer TMA with very little Atox1 expression in normal tissue (S1 Fig). For prognostic purposes, nuclear Atox1 expression was scored only in the cancer epithelium. We observed an increase in nuclear accumulations of Atox1 in epithelial cells in patients with stage IV metastatic CRC (p<0.0002; Fig 1). Nuclear location of Atox1 in the CRC patients is not associated with other clinicopathological variables such as gender, age and race. In contrast, the majority of Atox1 appeared in the cytoplasm of epithelial cells in non-metastatic stage I to III CRC patients. Thus, nuclear expression of Atox1 is significantly associated with disease severity and distant metastasis in CRC patients.

### Subcellular distribution of Atox1 protein in metastatic and non-metastatic CRC cell lines

**Fig 2:**
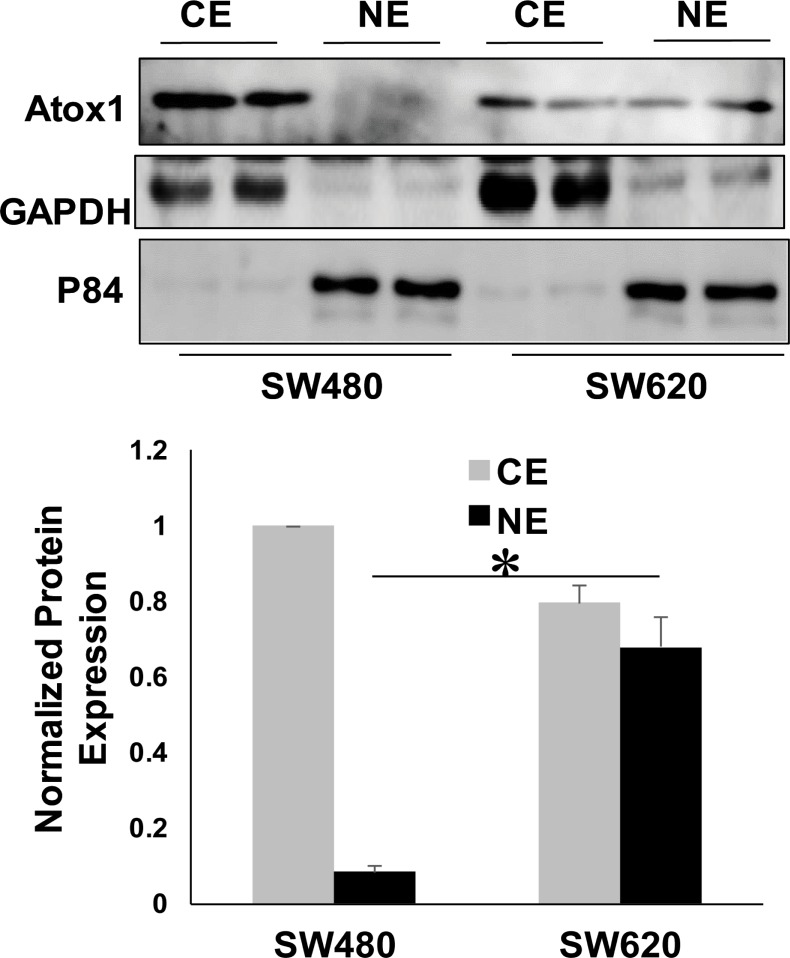
Metastatic SW620 CRC cells express higher levels of nuclear Atox1 than non-metastatic SW480 CRC cells. A, SW480 and SW620 colon cancer cells were subjected to subcellular fractionation followed by western blot analysis to determine the cellular localization of the Atox1 protein. GAPDH and Nuclear matrix protein p84 were used as markers for cytosolic and nuclear fractions, respectively. CE, cytoplasmic extract; NE, nuclear extract. Shown is a representative blot where samples were run in duplicate. B, Quantification by densitometry of the western blot results for Atox1 expression. Expression of Atox1 was normalized to the expression of or either GAPDH (CE) or p84 (NE). The CE expression was set at 1.0 and the NE Atox1 expression is expressed relative to the CE expression. *p < 0.05 compared to NE of SW480. Denistometry is averaged from 3 individual western blots.

The distinct nuclear localization of Atox1 protein in the colon cancer TMA prompted us to verify its subcellular distribution in CRC cell lines by cellular fractionation followed by immunoblotting. The nuclear localization of Atox1 protein was assessed in two cell lines derived from the same colon cancer patient. The SW480 cell line was derived from the primary colon tumor and is non-metastatic, while the SW620 colon cancer cell line was derived from the lymph node of the same patient when the cancer recurred with wide-spread metastases [24]. Efficient fractionation was confirmed by immunodetection of GAPDH in the cytoplasm and Nuclear matrix protein p84 in the nucleus (Fig 2). Consistent with TMA analysis, we observed higher levels of Atox1 protein in nuclear fractions of metastatic SW620 cells compared to non-metastatic SW480 cells. (Fig 2).

### Activin A-induced nuclear translocation of Atox1

**Fig 3:**
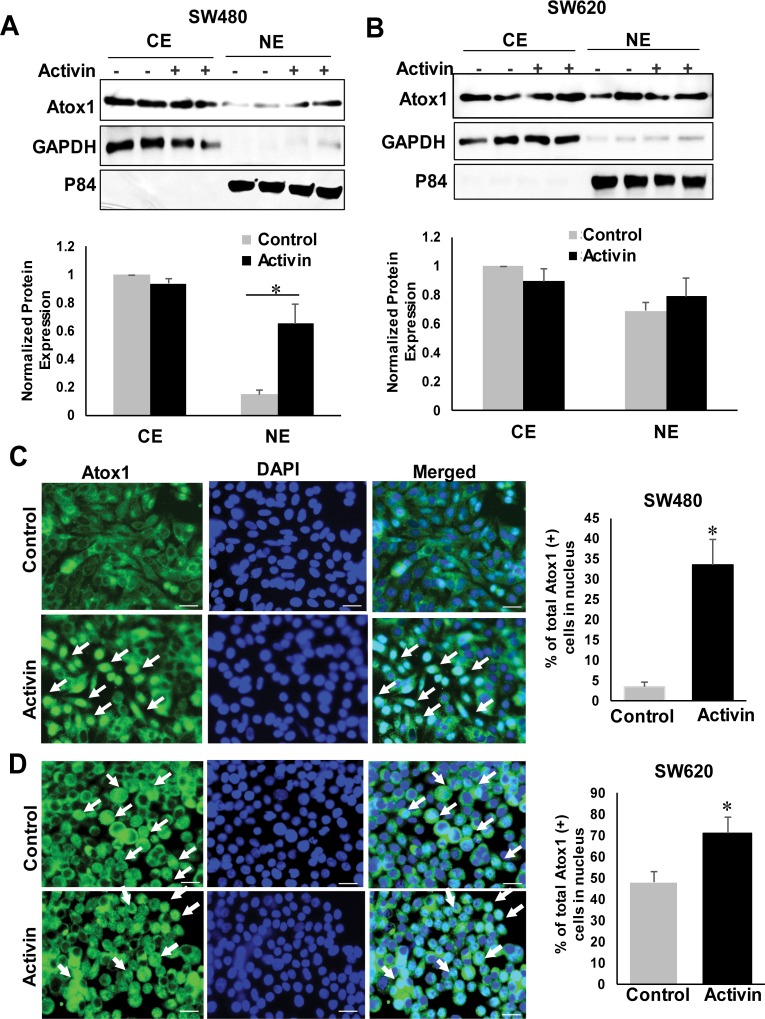
Activin A treatment increased the nuclear localization of Atox1 in colon cancer cells. Colon cancer cells were either untreated or treated with activin A (25 ng/ml) for 24 hours. A and B, Subcellular fractionation was followed by western blotting of lysates from untreated- and activin A-treated SW480 (A) and SW620 (B) colon cancer cells. GAPDH and p84 were used as markers for cytosolic and nuclear fractions. CE, cytoplasmic extract; NE, nuclear extract. Lower panel shows densitometric analysis of Atox1 protein levels. Results were normalized to GAPDH for cytoplasmic fraction and to p84 levels for nuclear fractions in the same experiments. *p < 0.05 versus untreated NE. C and D Immunofluorescence staining of Atox1. SW480 and SW620 cell were immunostained for Atox1 or nuclear marker DAPI. White arrow indicates nuclear Atox1. Scale bar 20μm. Data represent means + SD. *P _ 0.05.

To investigate the fate of Atox1 localization after activin A stimulation, SW480 and SW620 cells were treated with or without activin A for 24 hours. Effect of Activin A was assessed by subcellular fractionation (Fig 3A and 3B) and immunofluorescence analysis (Fig 3C and 3D). Activin A treatment induced an increase in nuclear Atox1 in the SW480 cells (Fig 3A and 3C). SW620 cells have high endogenous levels of Atox1 in the nucleus, which was increased further by activin A treatment (Fig 3B and 3D). Cytoplasmic levels of Atox1 did not change with activin A treatment in either cell line.

### Silencing of endogenous Atox1 decreased cyclin D1, p47 phox expression and inhibited cellular migration and colony formation in a metastatic CRC cell line

**Fig 4:**
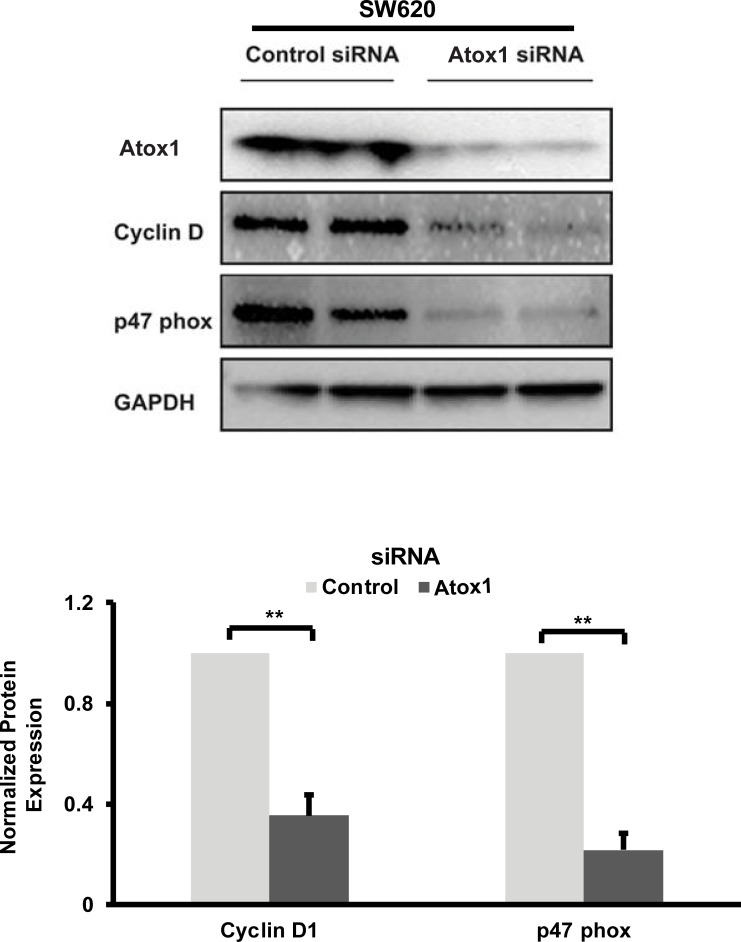
Knock-down of Atox1 decreases expression of cyclin D1 and NADPH oxidase p47 phox in CRC cells. A, Knock-down efficiency in SW620 metastatic CRC cells was confirmed by western blotting with and anti- Atox1 antibody. In addition, the Atox1 transcription targets of Cyclin D1 and p47 phox were measured by immunoblotting. The respective protein levels were quantified by densitometry scanning of immunoblots. Results were normalized to GAPDH expression in the same experiments. The experiments were repeated three times. * ** p < 0.01 Statistical analysis was performed by Student's t-test.

To further investigate the mechanism by which Atox1 may regulate metastasis in CRC, the endogenous expression of Atox1 was depleted by Atox1 siRNA in the metastatic SW620 cell line (Fig 4A). Since cyclin D1 acts as a transcriptional regulator of cell proliferation and migration [25], and Atox1 is known to induce cyclin D1 transcription [18, 26], we hypothesized that Atox1 could positively regulate colon cancer metastasis by influencing cyclin D1 expression. Following Atox1 knock-down (KD), cyclin D1 protein levels were significantly reduced (Fig 4).

Recent evidence has suggested a positive role for reactive oxygen species (ROS) in regulating EMT in cancer cells through influencing extracellular matrix remodeling, cytoskeleton remodeling, cell-cell junctions and cell mobility [27–29]. Therefore, we investigated the effect of endogenous Atox1 on the expression of NADPH oxidase, a major source of ROS in cancer [30] by measuring expression of p47 phox, a subunit of NADPH oxidase as a surrogate for ROS levels. KD of Atox1 significantly reduced p47 phox expression like cyclin D1 reduction under the same experimental conditions (Fig 4).

**Fig 5:**
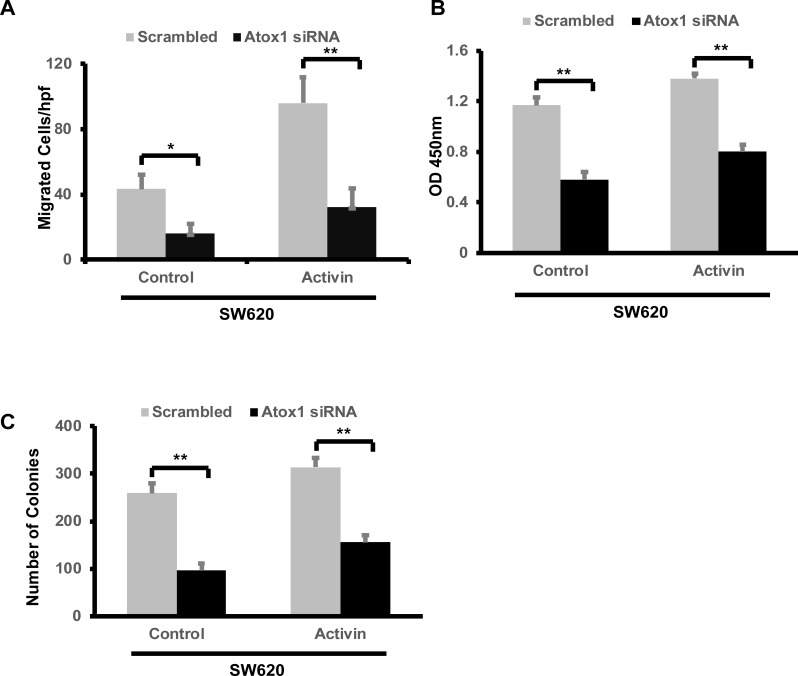
Knock-down of Atox1 in SW620 cells decreased cell migration and colony formation. A, CRC metastatic SW620 cell line was untreated or treated with activin A (25 ng/ml) for 6 hours under serum-free conditions following knock-down of endogenous Atox1. The cells were analyzed by transwell assay for migration and data are expressed as number of particles per high powered field (hpf). B, SW620 cells were treated as indicated above. Cell number relative to metabolic activity was assessed by CCK-8 assay after 18 hours of treatment. C, Following 24 hours of Atox1 knock-down, cells were divided and cultured in fresh complete media for 14 days. The colonies were fixed with cold methanol and stained with 0.05% of crystal violet and the number of colonies counted as described in methods. All the above experiments were repeated three times and results are mean SD of three independent experiments. *p <0.5 and**p < 0.01 respectively.

To explore whether nuclear accumulation of Atox1 has a functional consequence, we assessed cell migration of SW620 cells following Atox1 KD with and without activin A treatment. KD of Atox1 decreased SW620 cell migration when compared with cells transfected with scrambled siRNA. Interestingly, Atox1 KD attenuated activin A-induced cellular migration compared to scrambled control cells (Fig5A). KD of Atox1 also reduced SW620 cell number, as demonstrated by MTT assay (Fig 5B), which was not impacted by activin A treatment. As we previously reported [6, 7], activin A has very little impact on cell viability. Similar results were observed in a colony formation assay in which Atox1 KD reduced colony formation irrespective of activin A (Fig 5C, S2 Fig).

### Atox1 increased the metastatic potential of a non-metastatic CRC cell line

**Fig 6:**
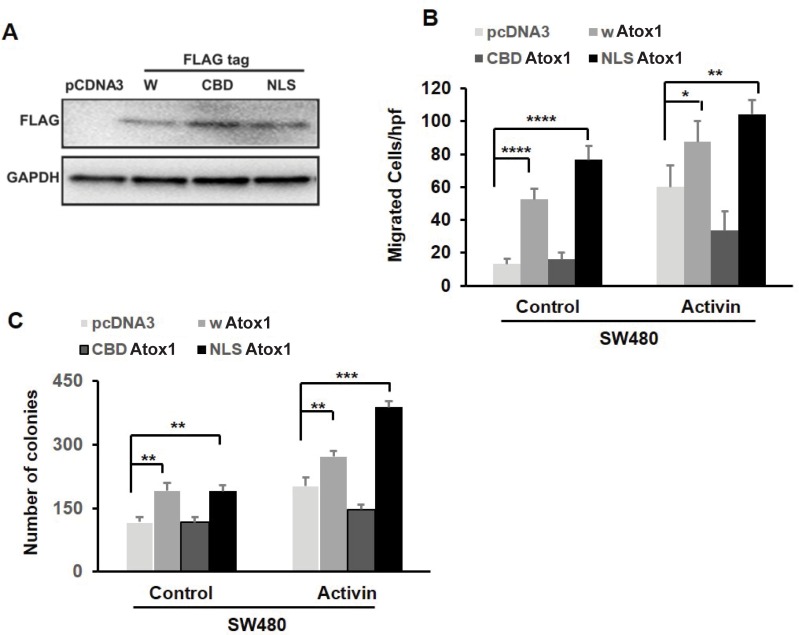
Over-expression of Atox1 in non-metastatic SW480 CRC cells increases cell migration and colony formation. A, CRC SW480 cells were transfected with either pCDNA3, FLAG-tagged wild type Atox1 (wATOX1), Cu-binding domain mutant (CBD) Atox1, or nuclear localization signal (NLS) Atox1 for 48 hours and transfection efficiency was confirmed by anti- FLAG antibody. B, Following 48 hours of transfection with the above plasmids, cells were cultured in fresh serum-free media for an additional 6 hours and migration was analyzed by transwell assay as described in methods. C, Colony formation assay was done under similar treatment condition with plasmids followed by an additional 14 days of culture in complete media. Colonies were stained with 0.05% crystal violet. All the above experiments were repeated three times and results are mean SD of three independent experiments. *p < 0.05, **p <0.01, ***p < 0.001 and **** p<0.0001 respectively.

Because our findings point to Atox1 as a positive regulator of metastasis, we over-expressed a nuclear-targeted Atox1 in the non-metastatic CRC cell line SW480 and assessed metastatic potential. To efficiently target Atox1 to the nucleus, an Atox1 nuclear localization signal (NLS) cDNA construct with a C-terminus Flag-tag was used as previously described [18]. In addition, we utilized a Flag-tagged wild type full length Atox1 construct (wtAtox1). A non-functional Atox1 construct with a mutated copper binding domain (CBD Atox1) and pcDNA3 empty vector were also used. The protein expression of the different constructs of Flag-tagged Atox1 transfected into SW480 cells is shown in Fig 6A. We performed a transwell migration assay with the transfected SW480 cell lines. The wATOX and NLS Atox1-transfected cells exhibited an increase in migratory activity compared to control vector-transfected cells, and activin A treatment further increased cell migration in these cell lines (Fig 6B). We have previously shown that the CBD of Atox1 is required for nuclear translocation[12]. The CBD Atox1 transfected cells did not have a change in cell migration compared to control vector-transfected cells. Moreover, CBD Atox1 cells did not respond to activin A treatment, suggesting the importance of the CBD of Atox1 for activin A-induced cell migration (Fig 6B).

The wAtox and NLS Atox1 cell lines also had increased colony formation compared to control vector cells, which again were further increased by activin A treatment. Colony formation was not changed in CBD Atox1 cell lines (Fig 6C, S3 Fig). These results indicate that the nuclear translocation of Atox1 protein is important for cellular migration and colony formation.

## Discussion

Atox1 is a copper (Cu) chaperone protein involved in maintaining copper homeostasis[31]. In addition to this role, we have previously demonstrated that Atox1 translocates to the nucleus upon copper binding where it can act as a transcription factor promoting growth by binding to the cyclin D1 promoter [18]. In line with its growth-promoting abilities, Atox1 is active in wound healing. Copper is present in higher concentrations in cancer tissues in comparison to normal tissues, and recent studies indicate that Atox1 may play a role in promoting carcinogenesis and cancer cell growth through increased reactive oxygen species (ROS)[16] and also increased cancer cell migration in indicated by the accumulation of Atox1 at lamellipodia borders of migrating breast cancer cells[14]. In this study we observed increased nuclear accumulation of Atox1 in metastatic CRC epithelial cells of patients indicating a potential transcriptional role for Atox1 in metastatic CRC. Using CRC cell lines developed from non-metastatic colon cancer tumor and from a lymph node metastasis from the same patient, we investigated the potential role of nuclear Atox1 in CRC and its regulation by activin A, a cytokine known to play a role in metastatic CRC[5].

In the metastatic SW620 cells, there is more Atox1 in the nucleus compared to the non-metastatic SW480 cells. In line with the nuclear enrichment of Atox1 in the SW620 cell, there is Atox1-dependent expression of growth promoting cyclin D1 and NADPH oxidase p47 phox subunit indicative of increased ROS accumulation. Treatment with activin A stimulates translocation of Atox1 into the nucleus of SW480 cells as well as inducing a further increase in nuclear Atox1 in SW620 cells. The functional significance of nuclear Atox1 was examined first in SW620 cells where knock-down of Atox1 expression resulted in decreased cell migration and colony formation which was unaffected by activin A. Further, we demonstrate that Atox1 knockdown by siRNA induced the most prominent proliferation suppression in metastatic SW620 colon cancer cell lines. This inhibitory effect was associated with inhibition of intracellular signaling as revealed by a decrease in cyclin D1 protein expression. In contrast, transfection of wild type Atox1 or nuclear localizing Atox1 into non-metastatic SW480 cells resulted in increased cell migration and colony formation which was further enhanced by activin A. The Cu-binding domain mutated Atox 1 was unable to increase either cell migration or colon formation and interestingly, decreased the activin A stimulation of those processes. This may indicate that Cu-bound Atox1 plays a role in the activin A stimulation of cell migration and colony formation. Although the presence of Atox1 in the nucleus has been reported in different cancers [13–17], this is the first demonstration that activin A stimulates cytoplasmic to nuclear transport of Atox1 leading to increased cellular migration and colony formation in non-metastatic colon cancer cell line. The nuclear import of proteins is highly regulated process and requires the presence of nuclear localization signal (NLS) at the carboxy terminus [32]. Given that Atox1 protein has a canonical NLS [18] our present study with transient transfection of nuclear-targeted Atox1 in non-metastatic SW480 cells strongly suggests that it is the nuclear Atox1 that plays an essential role in increased cellular migration and colony formation. These observations do not preclude that there may also be a role for cytoplasmic Atox1 in these processes. A decrease in cellular migration was observed when the copper binding domain was mutated, suggesting that Atox1 mediated cell migration and colony formation is Cu dependent. Previous studies have shown that loss of the Atox1 copper binding domain inhibits nuclear translocation [18]. Interestingly, we observed Atox1 cytoplasmic sequestration in SW480 colon cancer cell line and this restraint was released upon activin A stimulation that induced cytoplasmic to nuclear transport. Further investigation is necessary to decode the mechanisms involved in the cytoplasmic to nuclear trafficking of Atox1 in response to activin A stimulation. Given the strong increase of Atox1 protein levels in the nuclear fraction, changes in Atox1 driven transcription in response to activin A stimulation requires additional investigation.

### Conclusions

We report that Atox1 is localized both in cytoplasm and nucleus and its nuclear localization is enriched in metastatic CRC where it influences cell migration and colony formation. Activin A, an inflammatory cytokine increased in metastatic CRC, increases the nuclear localization of Atox1. In addition, Cu-bound Atox1 appears to enhance activin A-induced cell migration. Taken together, understanding the combined actions of activin A and Atox1 may improve the current staging of colorectal cancer patients.

## Supporting information

S1 Fig Atox1 staining of normal colon tissue. The adjacent normal colon tissue on the colon cancer tissue microarray was also stained for DAPI (blue) and Atox1 (red). Shown in a representative image indicating very little staining for Atox1 in normal colon tissue.
(EPS)

S2 Fig Reduction of Atox1 results in reduced colony formation. Image of SW620 colon cancer cell colonies formed after treatment with either scrambled or Atox1 siRNA and an additional treatment with either 25 ng/ml of activin or control PBS (Con). The number of colonies were quantified as indicated in the methods and show in Fig 5. Magnification of colonies was 2X.
(EPS)

S3 Fig Increased expression of Atox1 increases colony formation. Image of SW480 colon cancer cell colonies formed after transfection with the indicated vectors and an additional treatment with either 25 ng/ml of activin or control PBS (Con). The number of colonies were quantified as indicated in the methods and shown in Fig 6. Magnification of colonies was 10X.
(EPS)

S1 File File of western blots. This is the file of complete western blots used to created Fig 2, Fig 3 and Fig 4.
(PDF)
