# Supplementary figures and images for: Nuclear translocation of Atox1 potentiates activin A-induced cell migration and colony formation in colon cancer
Source: PLoS One. 2020 Jan 21;15(1):e0227916. doi: 10.1371/journal.pone.0227916 (PMC6974162; doi:10.1371/journal.pone.0227916)

Figure 2

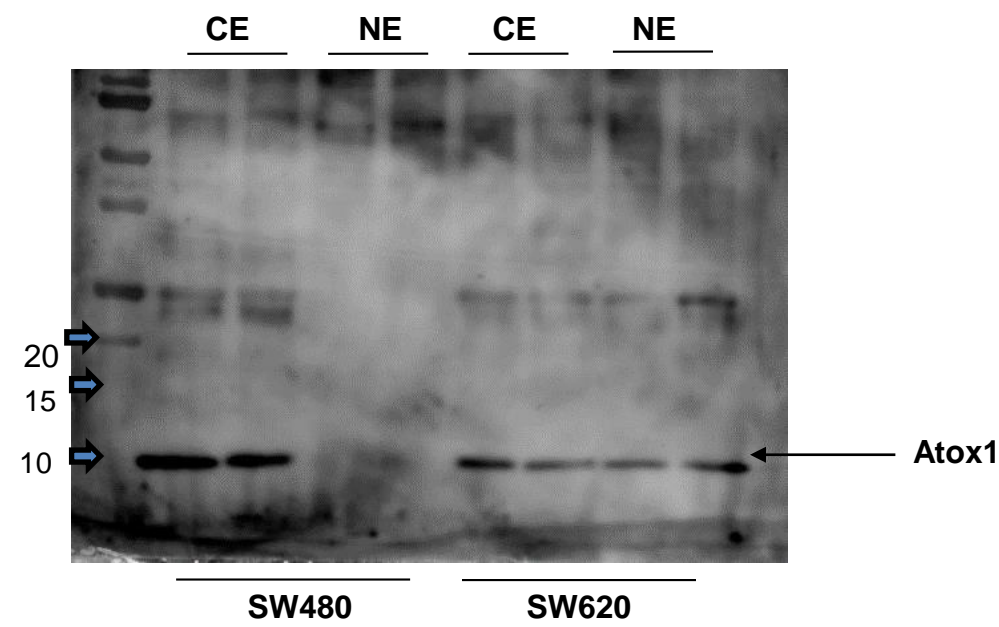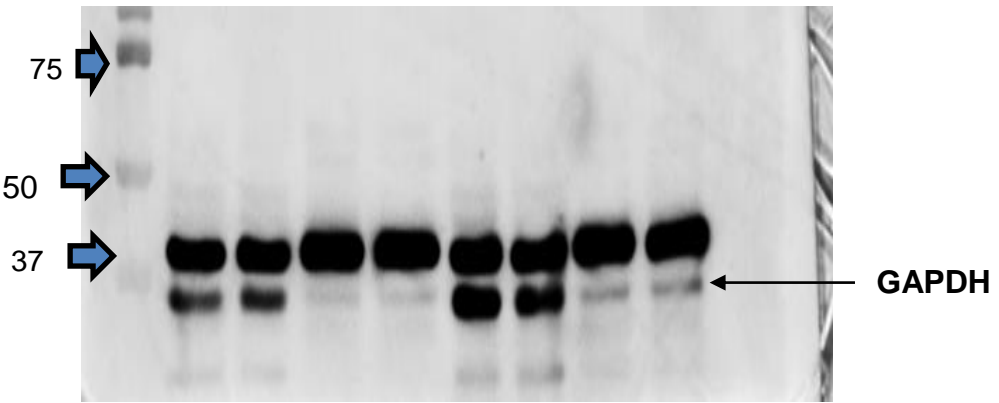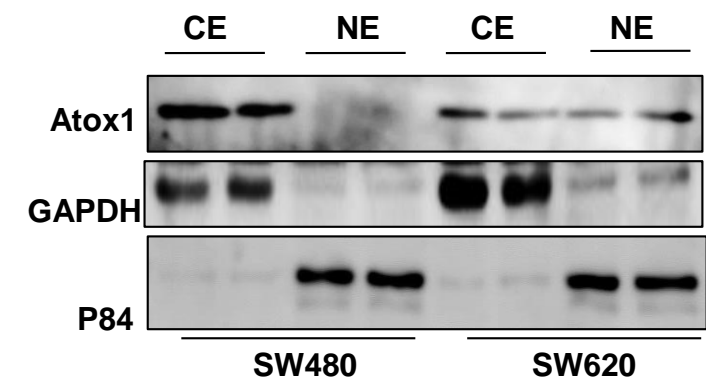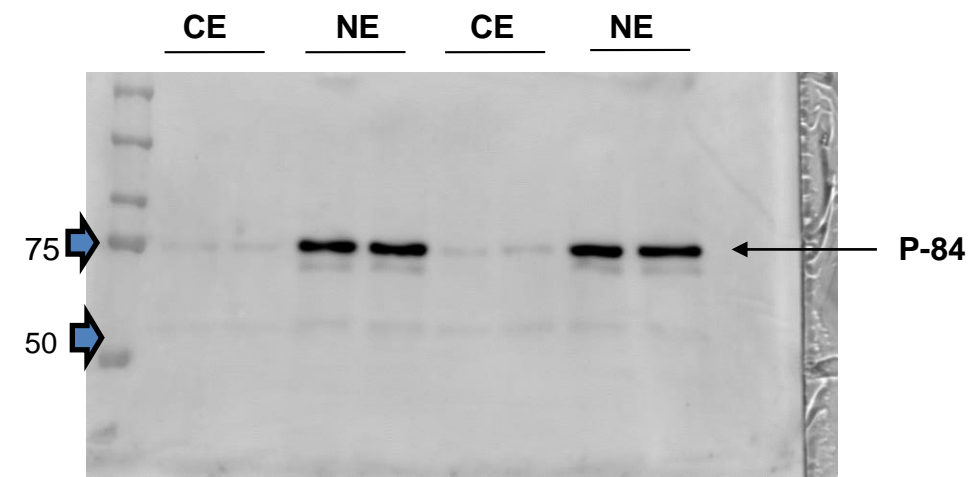

Figure 3B, SW620

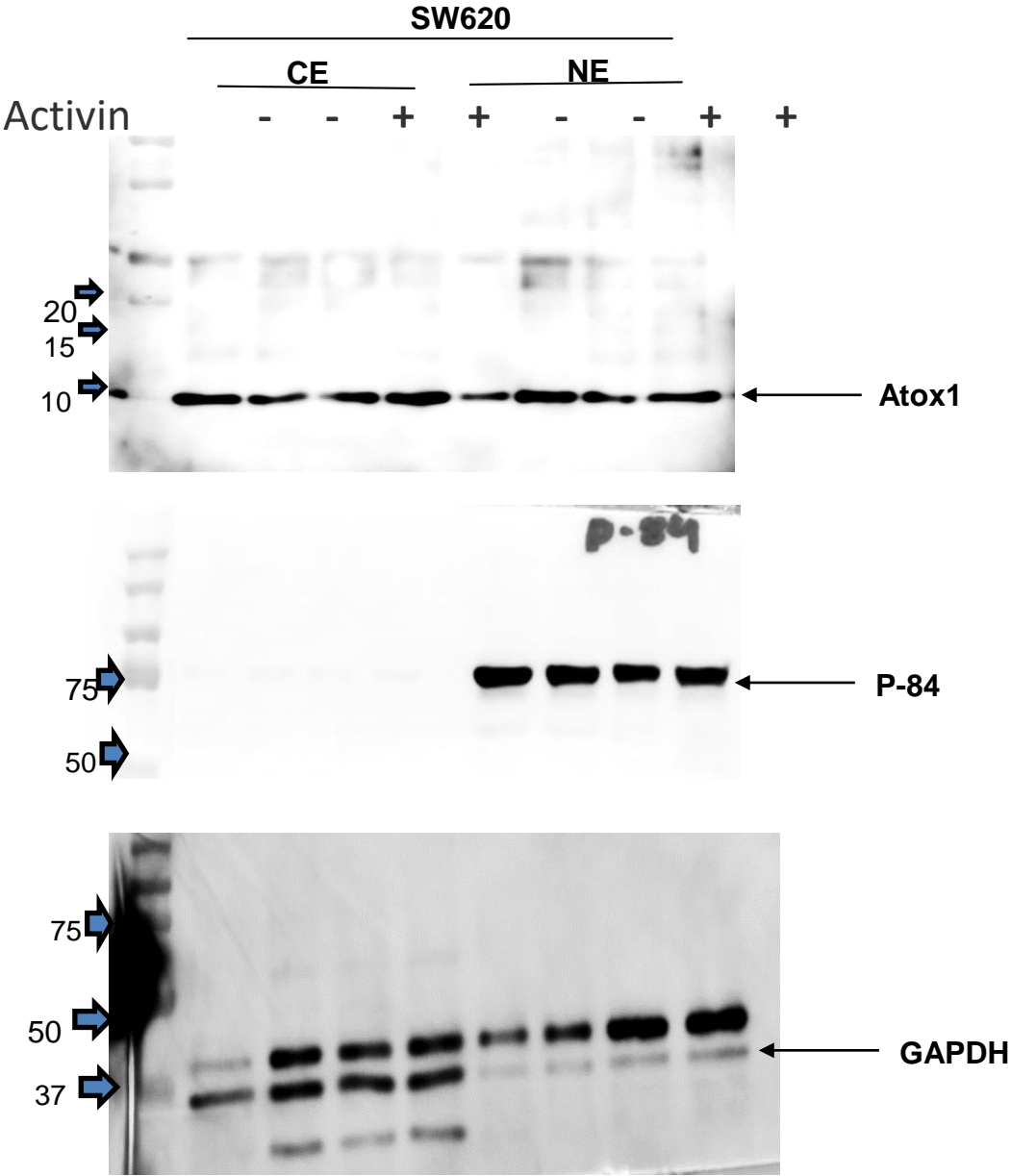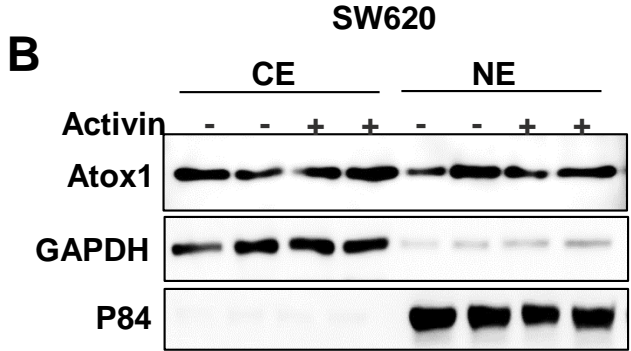

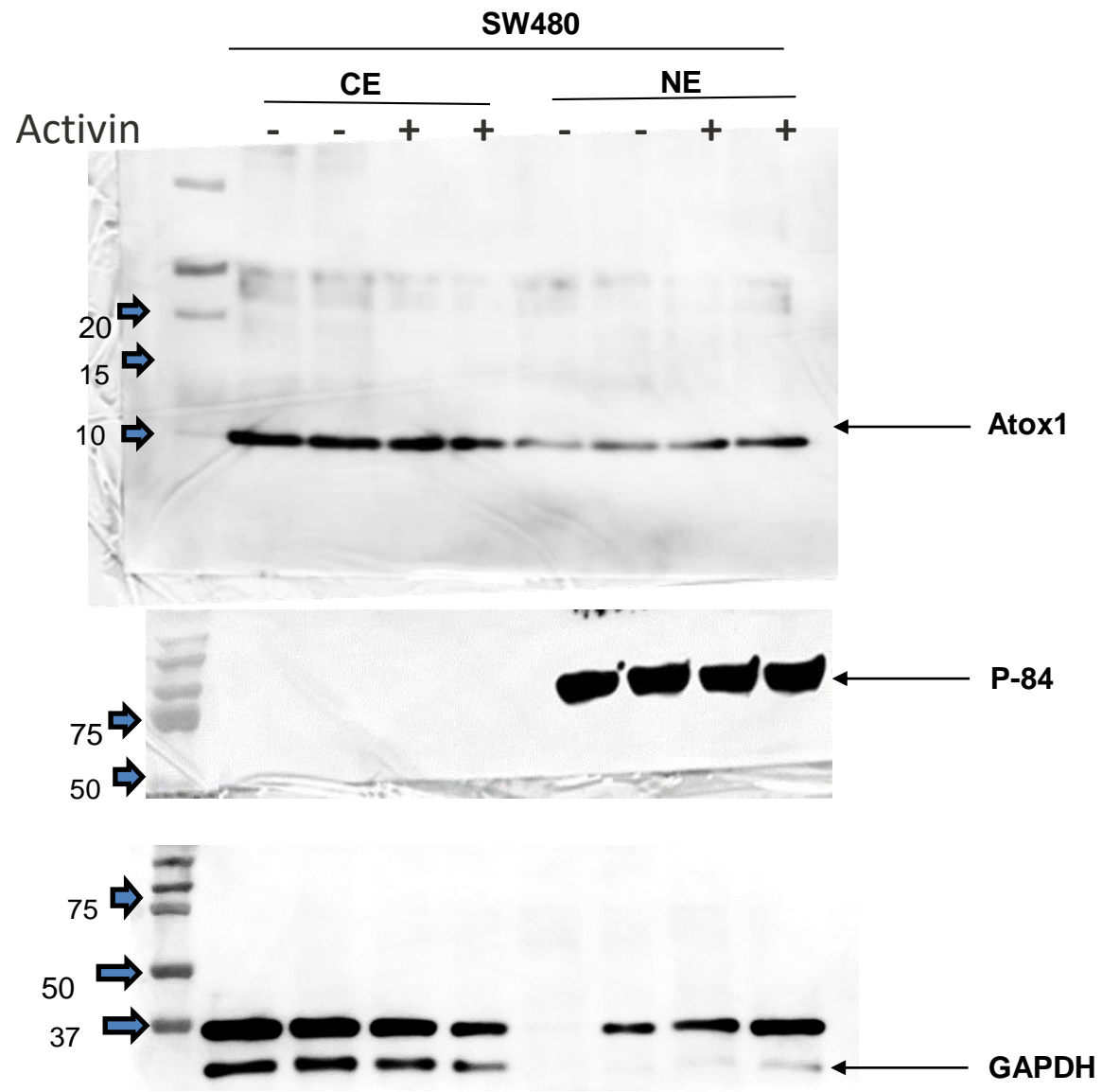

Figure 3A, SW480

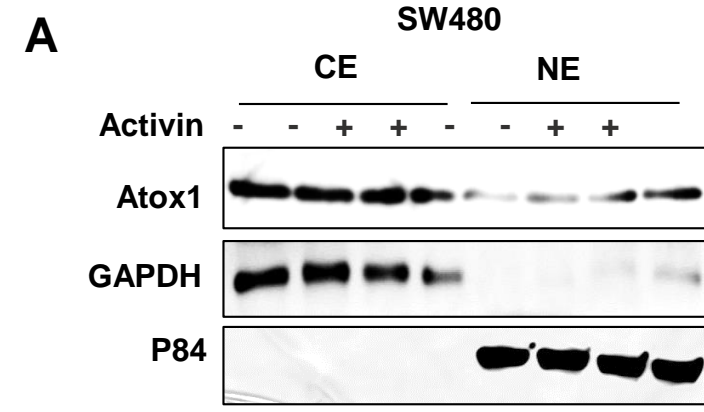

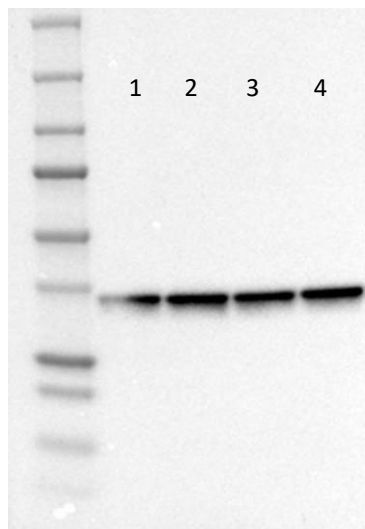

**GAPDH**

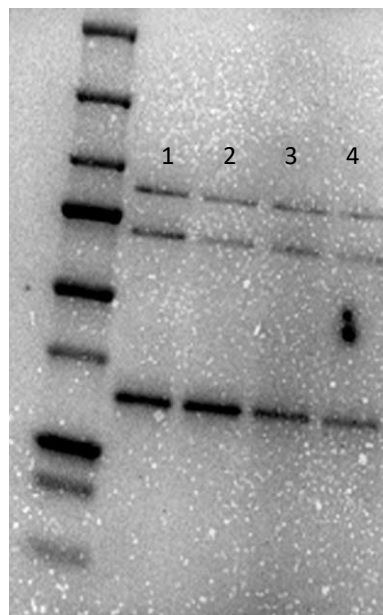

**Cyclin D**

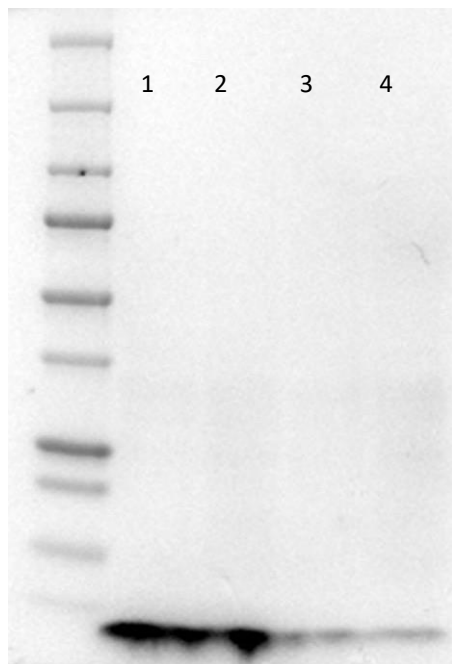

**Atox1**

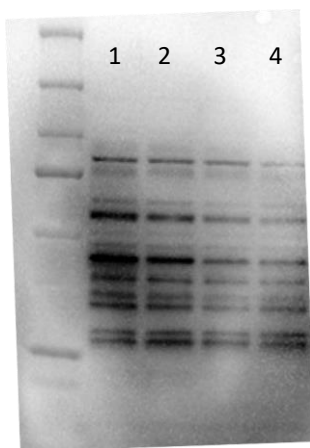

**P47 phox**

Figure 4

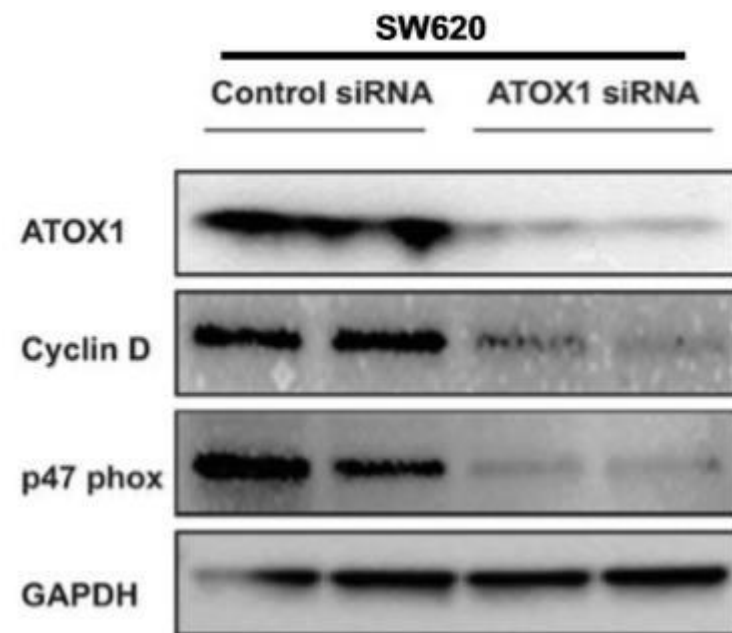

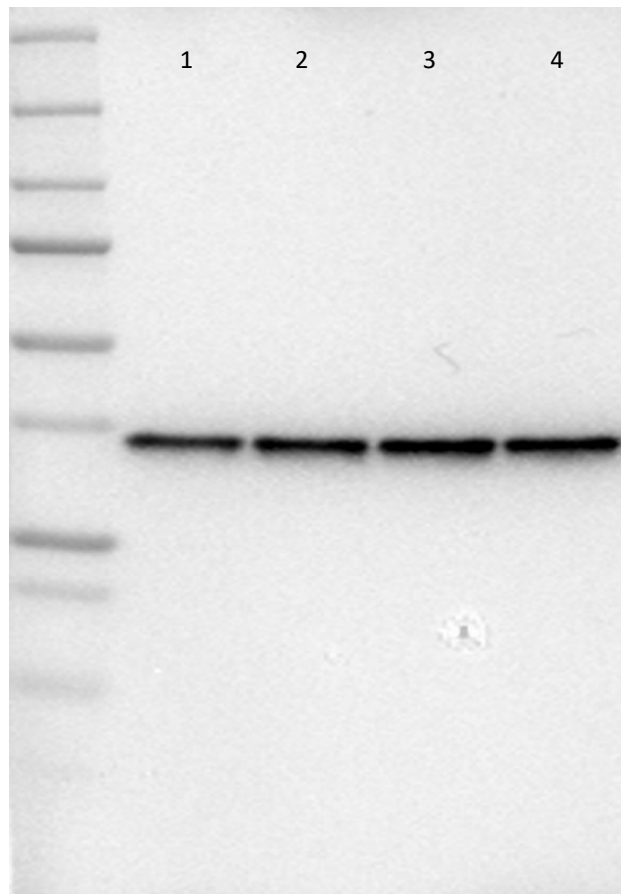

GAPDH

**A**

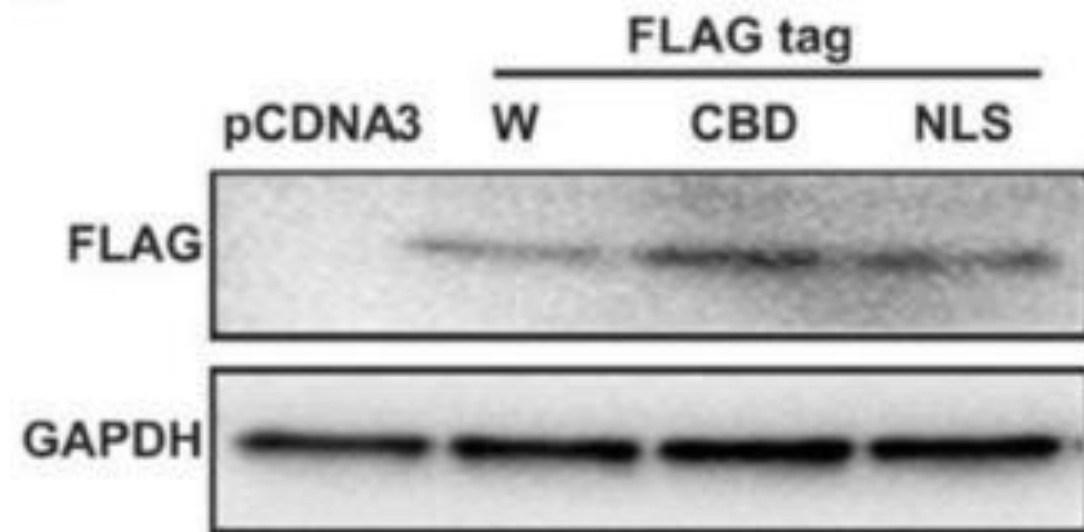

Figure 6

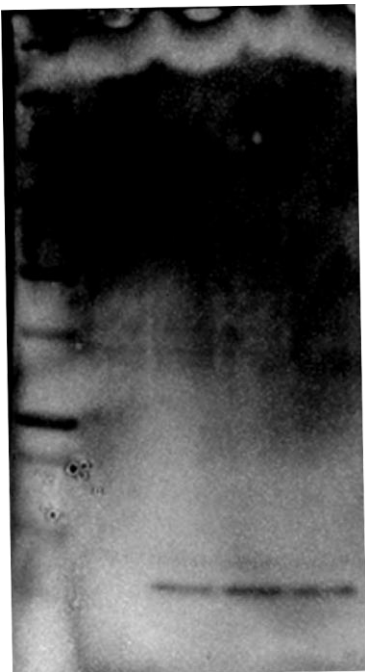

FLAG

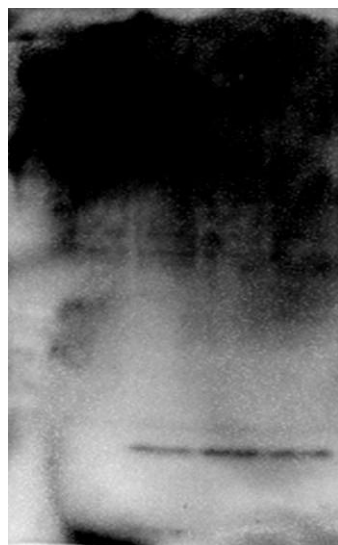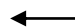

1 2 3 4

FLAG

Supplement: S1 File — This is the file of complete western blots used to created Fig 2, Fig 3 and Fig 4. (PDF) [file pone.0227916.s004.pdf]
